# Metastatic Paraganglioma With a Fatal Outcome Following Liver Biopsy: A Case Report

**DOI:** 10.7759/cureus.90730

**Published:** 2025-08-22

**Authors:** Isabel Hipólito-Reis, Núria Jorge, Filipe Cruz, Sara Nogueira, José-Artur Paiva

**Affiliations:** 1 Department of Intensive Care Medicine, Centro Hospitalar Universitário de São João, Porto, PRT; 2 Department of Surgery, Centro Hospitalar Universitário de São João, Porto, PRT; 3 Department of Anaesthesiology, Centro Hospitalar Universitário de São João, Porto, PRT; 4 Department of Medicine, Faculty of Medicine, University of Porto, Porto, PRT

**Keywords:** catecholaminergic crisis, eus-guided liver biopsy, extra-adrenal paraganglioma, hemorrhagic shock, irreversible ischemia, metastatic paraganglioma, multiorgan system failure

## Abstract

Paragangliomas are rare neuroendocrine tumors that may present as hormonally active or silent masses. While generally indolent, they may metastasize and lead to severe complications, particularly when biopsied or manipulated without prior diagnosis. We describe a 53-year-old woman with a history of hypertension, diabetes, and dyslipidemia, admitted for investigation of constitutional symptoms and lytic vertebral lesions. Imaging revealed liver and bone metastases, a retroperitoneal mass, and adenopathy. A liver biopsy was performed, after which she developed hemorrhagic shock due to active hepatic bleeding. Despite successful embolization, she progressed to multiorgan failure, extensive intestinal ischemia, and death. Histological analysis revealed metastatic paraganglioma. This case highlights the diagnostic and therapeutic challenges associated with occult, functionally active paraganglioma. Invasive procedures in undiagnosed neuroendocrine tumors may trigger life-threatening complications, especially in hormonally active lesions. A routine biochemical evaluation is recommended before biopsy, as early recognition of paraganglioma in retroperitoneal masses, even when asymptomatic, is essential to prevent fatal outcomes.

## Introduction

Paragangliomas are rare neuroendocrine tumors, with a prevalence between 1:2,500 and 1:6,500 [1]. These tumors arise from chromaffin cells, most commonly located in the adrenal medulla (pheochromocytomas) or extra-adrenal sites. Although many are benign, approximately 10-20% may metastasize, most often to bone, liver, lungs, or lymph nodes [2].

Functional paragangliomas can secrete catecholamines, leading to classic symptoms such as paroxysmal hypertension, tachycardia, diaphoresis, and weight loss. In contrast, non-functional tumors may remain clinically silent, delaying recognition until advanced disease or being discovered incidentally on imaging. Metastatic disease often presents with non-specific symptoms, including bone pain, constitutional features, or organ-specific dysfunction, which may overlap with more common conditions and contribute to diagnostic delay [3,4].

The differential diagnosis of metastatic paraganglioma includes other neuroendocrine tumors, adrenal cortical carcinoma, renal cell carcinoma, and metastatic lesions from primary lung or gastrointestinal malignancies [5]. The diagnosis is typically established by biochemical assays (plasma or urine metanephrines) combined with anatomical and functional imaging, such as CT/MRI and PET with specific radiotracers (e.g., 18F-fluorodeoxyglucose, 18F-fluorodopa, and 68Ga-DOTATATE) [6]. Histological confirmation may be necessary in select cases, but biopsy is generally avoided due to the risk of catecholamine surge or hemorrhage [7].

Management of metastatic paraganglioma is challenging and often requires a multidisciplinary approach. Treatment options include surgical resection when feasible, radionuclide therapy (e.g., 131 I-MIBG or 177Lu-DOTATATE), external-beam radiotherapy, systemic chemotherapy, and targeted agents such as tyrosine kinase inhibitors [8,9]. Despite these options, disease progression is common, and complications may arise from both tumor burden and catecholamine excess, including refractory hypertension, arrhythmias, cardiomyopathy, or multiorgan dysfunction [10].

This case report describes a patient in whom a paraganglioma was not suspected prior to biopsy, with the diagnosis only established after a fatal post-biopsy course.

## Case presentation

A 53-year-old woman, with a Clinical Frailty Scale score of 4 (moderately frail, some dependence for activities of daily living), with medical history of long-standing hypertension (on four antihypertensive agents), type 2 diabetes mellitus, and dyslipidemia, presented to the emergency department with complaints of persistent left lower back pain, anorexia, unintentional weight loss, and profuse night sweats over one month. She had no known drug allergies. Her cancer screening was up to date. These clinical features, particularly refractory hypertension and the new or worsening manifestation of diabetes mellitus, are classic indicators of catecholamine-secreting tumors and could support earlier biochemical investigation.

A recent lumbar CT scan had shown osteolytic vertebral lesions. Blood tests revealed acute kidney injury (serum creatinine = 3.24 mg/dL) and elevated liver enzymes (aspartate aminotransferase = 1825 U/L; alanine aminotransferase = 1547 U/L). A new thoracoabdominal-pelvic CT scan showed a heterogeneous retroperitoneal solid mass without extension to the pancreas or adrenal glands, retroperitoneal lymphadenopathy, and extensive hepatic and bone involvement, suggestive of metastases.

She was admitted for further investigation. Serum protein electrophoresis and tumor markers were unremarkable. On day six, an ultrasound-guided liver biopsy was performed without immediate complications.

Approximately four hours post-biopsy, the patient developed hypotension (94/50 mmHg), sinus tachycardia (170 bpm), and signs of peripheral hypoperfusion. An urgent CT angiogram revealed active bleeding at the liver biopsy site. She was transferred to a tertiary hospital for interventional radiology, where hepatic embolization was successfully performed, and admitted to the intensive care unit (ICU).

**Figure 1:**
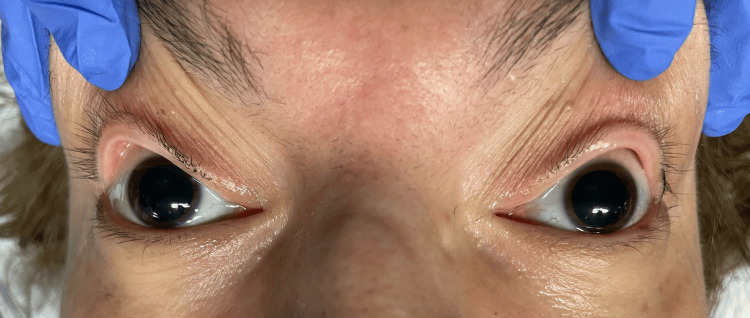
Dilated pupils at presentation.

On ICU arrival, she was hypotensive (80/67 mmHg), with metabolic acidosis (pH = 7.00), a lactate level of 13 mmol/L, and a Glasgow Coma Scale score of 10. Her pupils were fixed and dilated (Figure 1). She was then intubated and mechanically ventilated. Her hemoglobin level was 7 g/dL, and her glucose level was 600 mg/dL. She received 1 g of tranexamic acid, three units of packed red blood cells, intravenous fluids, and bicarbonate. Blood gases after initial resuscitation showed some improvement (pH = 7.28, bicarbonate (HCO₃⁻) = 14 mmol/L).

**Figure 2:**
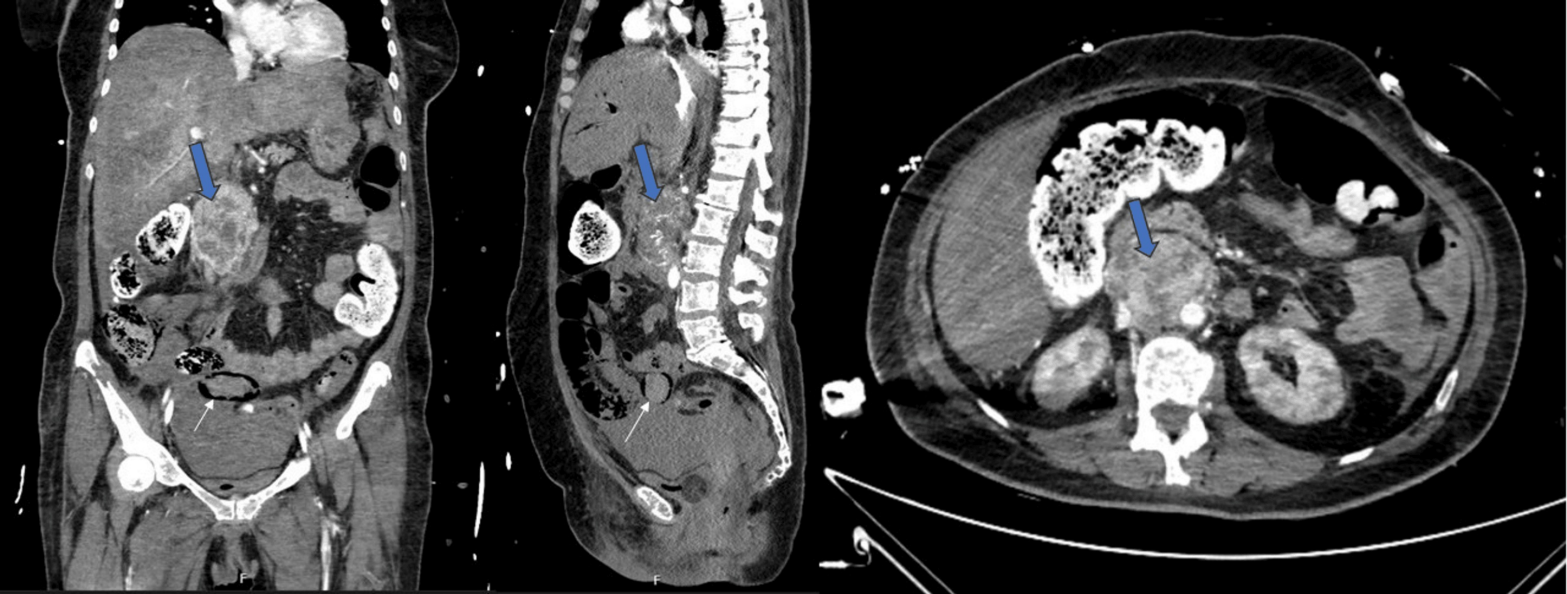
Coronal, sagittal, and axial CT images in the portal venous phase. Coronal (left), sagittal (center), and axial (right) portal venous phase CT images of the thoracoabdominopelvic region. The blue arrow identifies a heterogeneous retroperitoneal mass. The thin white arrow indicates intestinal pneumatosis.

Over the following hours, she developed persistent high-grade fever despite triple antipyretic therapy, worsening renal and hepatic function, and required increasing doses of noradrenaline. Repeat CT (Figure 2) showed the previously described heterogeneous retroperitoneal mass, and additionally revealed hemoperitoneum without active bleeding, portal venous gas, extensive pneumatosis intestinalis (notably in the ileum), and numerous gas bubbles in mesenteric and ileocolic venous branches. Splenic and renal ischemia were also suspected.

General surgeons evaluated the patient and deemed the extent of intestinal ischemia unrecoverable. Given irreversible multiorgan failure, a decision was made to provide palliative care. The patient died approximately 48 hours after ICU admission.

Post-mortem histopathological analysis of the liver biopsy revealed the diagnosis of metastatic paraganglioma.

## Discussion

Paragangliomas are often diagnosed late due to nonspecific symptoms or silent progression. Functional tumors may present earlier due to catecholamine secretion, but non-functional variants can remain undetected until metastatic spread or complications arise [4].

In this case, the retroperitoneal mass and bone metastases were suspicious for malignancy, but tumor markers were negative, and the misinterpretation of classic paraganglioma symptoms delayed suspicion. Importantly, liver biopsy in such tumors carries a high risk of bleeding and potentially a catecholaminergic crisis [11]. This patient developed hemorrhagic shock shortly after the biopsy, despite no immediate signs during the procedure.

The profound metabolic derangements, fixed mydriasis, and rapidly worsening multiorgan failure raise the possibility of a catecholamine surge, compounded by hemorrhage-induced hypoperfusion. Furthermore, extensive intestinal ischemia, gas in the portal circulation, and splenic/renal hypoperfusion confirmed irreversible shock.

Histological diagnosis of paraganglioma was only available post-mortem. This underscores the importance of early recognition. Imaging features of paragangliomas may include intense arterial enhancement, which shows hypervascularity and necrosis, particularly on contrast-enhanced CT or MRI. When retroperitoneal or adrenal masses are detected, biochemical screening with plasma metanephrines should precede biopsy [10,12].

## Conclusions

This case illustrates the serious consequences of unrecognized paraganglioma, particularly when invasive procedures are performed. In patients with retroperitoneal masses and lytic bone lesions, even in the absence of typical symptoms, paraganglioma should be considered in the differential diagnosis. We advocate for multidisciplinary pre-biopsy evaluation of retroperitoneal masses and routine screening for catecholamine excess in at-risk patients. Awareness of this rare but lethal complication may help prevent similar outcomes.

## References

[1] Aygun N, Uludag M (2020). Pheochromocytoma and paraganglioma: from epidemiology to clinical findings. Sisli Etfal Hastan Tip Bul.

[2] Fishbein L, Nathanson KL (2012). Pheochromocytoma and paraganglioma: understanding the complexities of the genetic background. Cancer Genet.

[3] Brewczyński A, Kolasińska-Ćwikła A, Jabłońska B, Wyrwicz L (2025). Pheochromocytomas and paragangliomas—current management. Cancers (Basel).

[4] Ayala-Ramirez M, Feng L, Johnson MM (2011). Clinical risk factors for malignancy and overall survival in patients with pheochromocytomas and sympathetic paragangliomas: primary tumor size and primary tumor location as prognostic indicators. J Clin Endocrinol Metab.

[5] Shah MH, Goldner WS, Benson AB (2021). Neuroendocrine and adrenal tumors, version 2.2021, NCCN clinical practice guidelines in oncology. J Natl Compr Canc Netw.

[6] Taïeb D, Hicks RJ, Hindié E (2019). European Association of Nuclear Medicine Practice Guideline/Society of Nuclear Medicine and Molecular Imaging Procedure Standard 2019 for radionuclide imaging of phaeochromocytoma and paraganglioma. Eur J Nucl Med Mol Imaging.

[7] Mete O, Asa SL, Gill AJ, Kimura N, de Krijger RR, Tischler A (2022). Overview of the 2022 WHO classification of paragangliomas and pheochromocytomas. Endocr Pathol.

[8] Sukrithan V, Perez K, Pandit-Taskar N, Jimenez C (2024). Management of metastatic pheochromocytomas and paragangliomas: when and what. Curr Probl Cancer.

[9] Zhang L, Åkerström T, Mollazadegan K, Beuschlein F, Pacak K, Skogseid B, Crona J (2023). Risk of complications after core needle biopsy in pheochromocytoma/paraganglioma. Endocr Relat Cancer.

[10] Lenders JW, Duh QY, Eisenhofer G (2014). Pheochromocytoma and paraganglioma: an endocrine society clinical practice guideline. J Clin Endocrinol Metab.

[11] Mannelli M, Lenders JW, Pacak K, Parenti G, Eisenhofer G (2012). Subclinical phaeochromocytoma. Best Pract Res Clin Endocrinol Metab.

[12] Pacak K, Eisenhofer G, Ahlman H (2007). Pheochromocytoma: recommendations for clinical practice from the First International Symposium. Nat Clin Pract Endocrinol Metab.

